# Supplementary material for: Guidance landscapes unveiled by quantitative proteomics to control reinnervation in adult visual system
Source: Nat Commun. 2022 Oct 13;13:6040. doi: 10.1038/s41467-022-33799-4 (PMC9561644; doi:10.1038/s41467-022-33799-4)
Supplement: Supplementary file 1 — Supplementary Information [file 41467_2022_33799_MOESM1_ESM.pdf]

Supplementary Figures for

Guidance landscapes unveiled by quantitative proteomics to control reinnervation in adult visual system

Noemie Vilallongue[1†], Julia Schaeffer[1†], Anne-Marie Hesse[2], Céline Delpech[1], Béatrice Blot[1], Antoine Paccard[1], Elise Plissonnier[1], Blandine Excoffier[1], Yohann Couté[2], Stephane Belin[1‡], Homaira Nawabi[1*‡]

† These authors contributed equally.
‡ These authors jointly supervised this work.
*Email: homaira.nawabi@inserm.fr

This PDF file includes:

Supplementary Figures 1 to 7
Supplementary References

Supplementary Figure 1

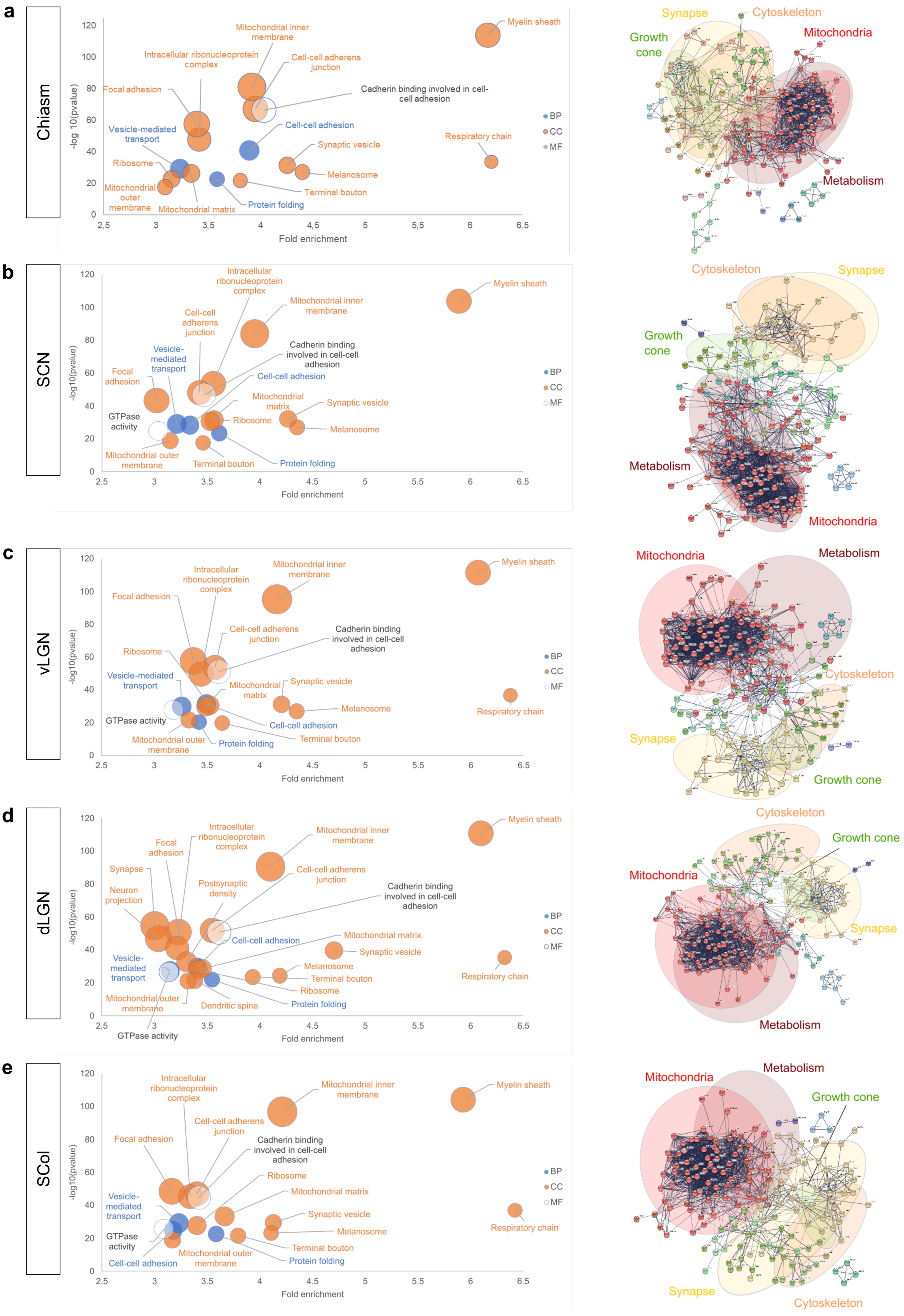

Supplementary Fig. 1 Adult visual targets exhibit enrichment of proteins related to connectivity and synaptic activity. a-e Bubble plots displaying enriched Gene Ontology (GO) terms in **a** optic chiasm, **b** SCN, **c** vLGN, **d**

dLGN and **e** SCol, as analyzed with DAVID (left). Enriched GO terms are related to myelin sheath (GO:0043209), mitochondrial constituents (eg, GO:0005759 mitochondrial matrix, GO:0005743 mitochondrial inner membrane, GO:0070469 respiratory chain) and synapses (eg, GO:0008021 synaptic vesicle, GO:0014069 postsynaptic density), all terms characteristic of neuronal activity. Also, some of the most enriched GO terms shared by all regions of interest are linked to cell adhesion (eg, GO:0005913 cell-cell adherens junction, GO:0005925 focal adhesion, GO:0098641 cadherin binding involved in cell-cell adhesion, GO:0098609 cell-cell adhesion), reflective of a high connectivity within the tissue. Terms associated with biological processes (BP) are represented in blue, cellular compartments (CC) in orange and molecular functions (MF) in grey. Only GO terms with a protein count higher than 50, a fold enrichment higher than 3 and a corrected p-value < 0.01 are represented. The p-value is the modified Fisher Exact p-value (EASE score). The bubble size of each GO term is representative of the number of proteins detected in the corresponding brain region. Network-based cluster analysis of the 200 most abundant proteins identified in each visual target (right): **a** optic chiasm, **b** SCN, **c** vLGN, **d** dLGN, **e** SCol as analyzed with STRING. Only interactions with high confidence (minimum required interaction score > 0.700) are represented. Clustering is done with a Markov Cluster Algorithm (MCL = 1.4). Corresponding functional categories were manually annotated. Source data are provided as a Source Data file.

Supplementary Figure 2

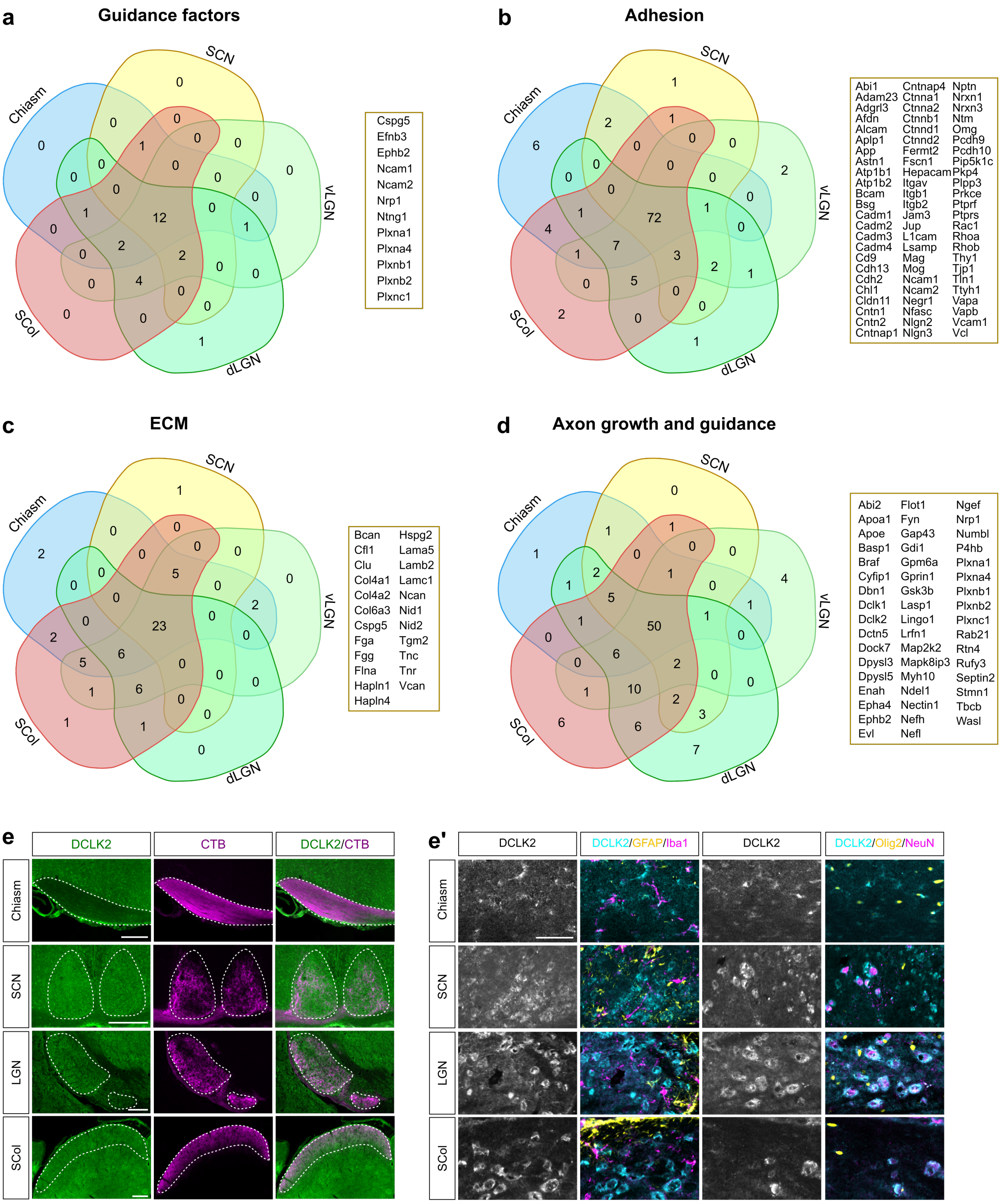

f

		GFAP	Iba1	Olig2	NeuN
DCLK2	Chiasm			+	
	SCN				++
	LGN				++
	SCol				++

Supplementary Fig. 2 Validation of proteomics analysis. a-d Venn diagram representing the shared **a** guidance factors hits, **b** adhesion-related hits, **c** extracellular-matrix-related hits, **d** axon growth and guidance-related hits (number of proteins and percentage of the protein number in the guidance factors, adhesion, extracellular-matrix and, axon growth and guidance categories as defined in **Supplementary Data 2**). In boxes are indicated proteins detected in all visual targets. **e** Epifluorescence images of DCLK2

immunofluorescent labelling in the adult chiasm, SCN, LGN and SCol (co-labelled with CTB). Scale bar: 200µm. **e'** Confocal images of DCLK2 and different cell populations markers (GFAP, Iba1, Olig2, NeuN) in the adult intact chiasm, SCN, LGN and SCol. Scale bar: 50µm. All images are representative of N=3 biologically independent animals. **f** Table summarizing the type of cells expressing the guidance molecules of interest.

Supplementary Figure 3

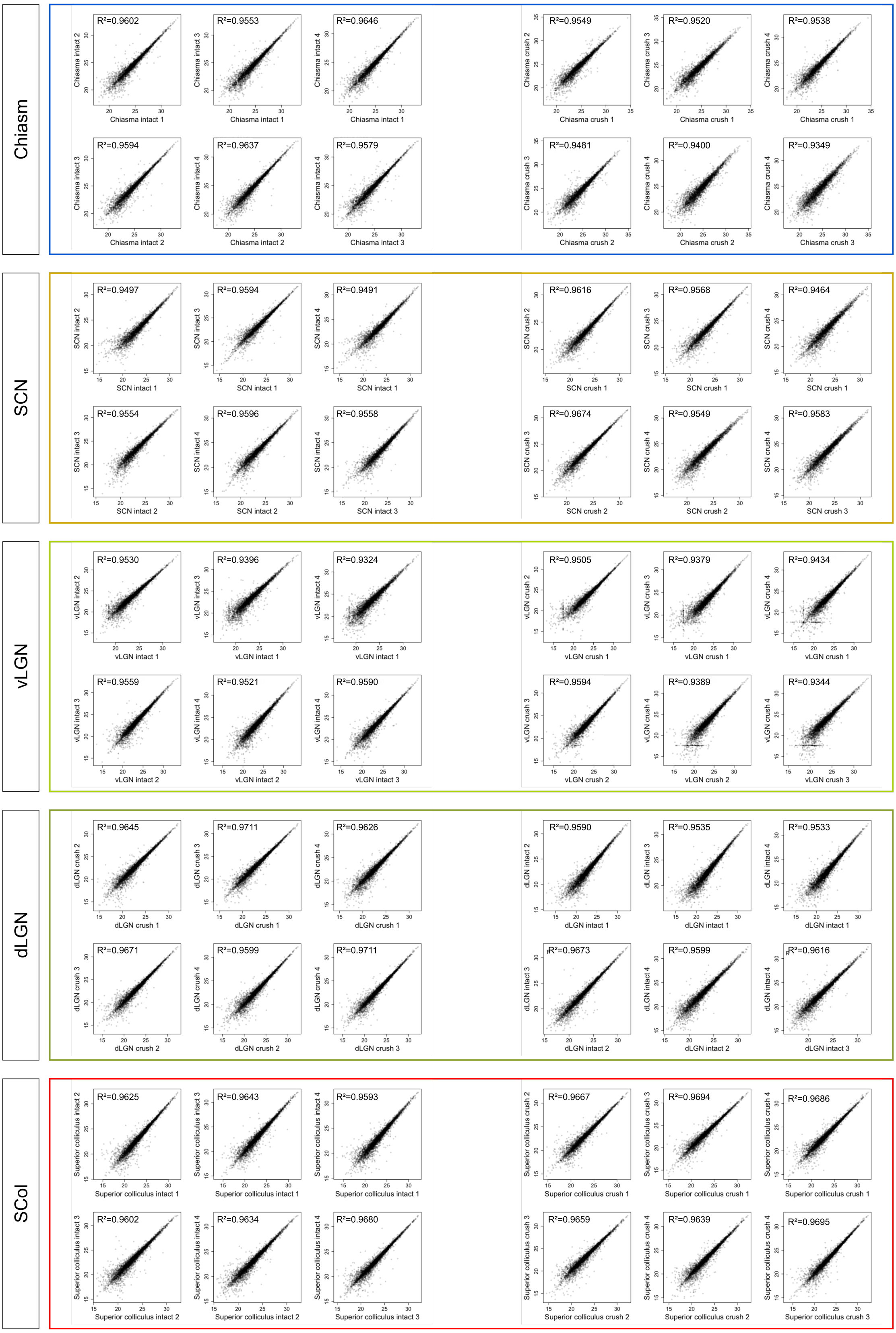

Supplementary Fig. 3 High reproducibility of the experimental design. Scatterplots of protein abundance of the detected hits across replicates, in intact (left) and injured (crush, right) conditions. The Pearson's correlation coefficient is indicated on each plot.

Supplementary Figure 4

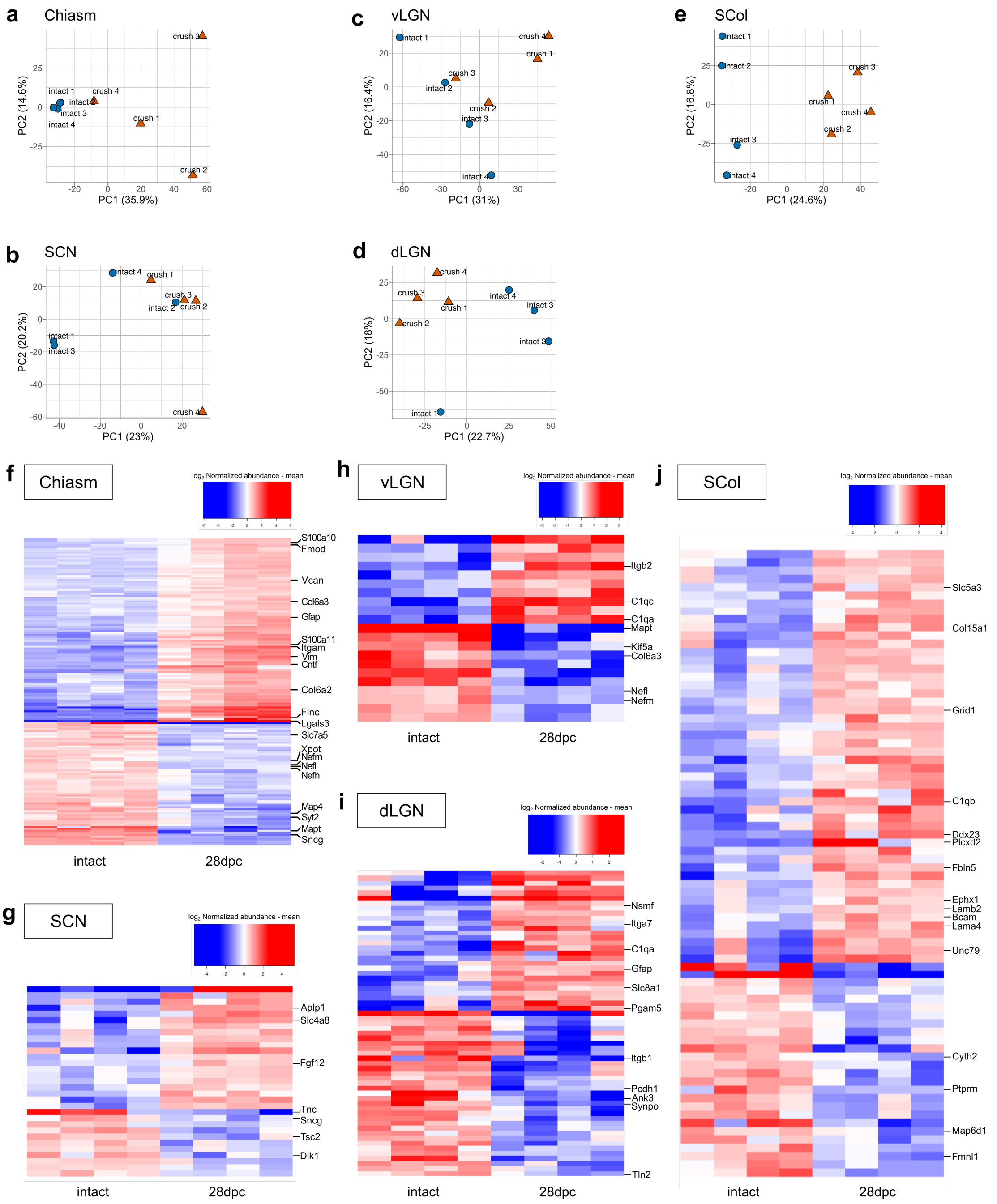

Supplementary Fig. 4 Bilateral optic nerve crush causes modifications of the proteome compared to intact. a-e PCA analysis showing a clustering of the replicates according to the condition intact or injured (crush) in each adult visual target. **f-j** Heatmaps showing differentially expressed proteins between injured (crush) and intact conditions. Values are the difference between log_2 normalized abundance of each protein hit and the mean across all samples. For the optic chiasm, proteins with FDR < 1% are represented. For other targets, proteins with FDR < 5% are represented.

Supplementary Figure 5

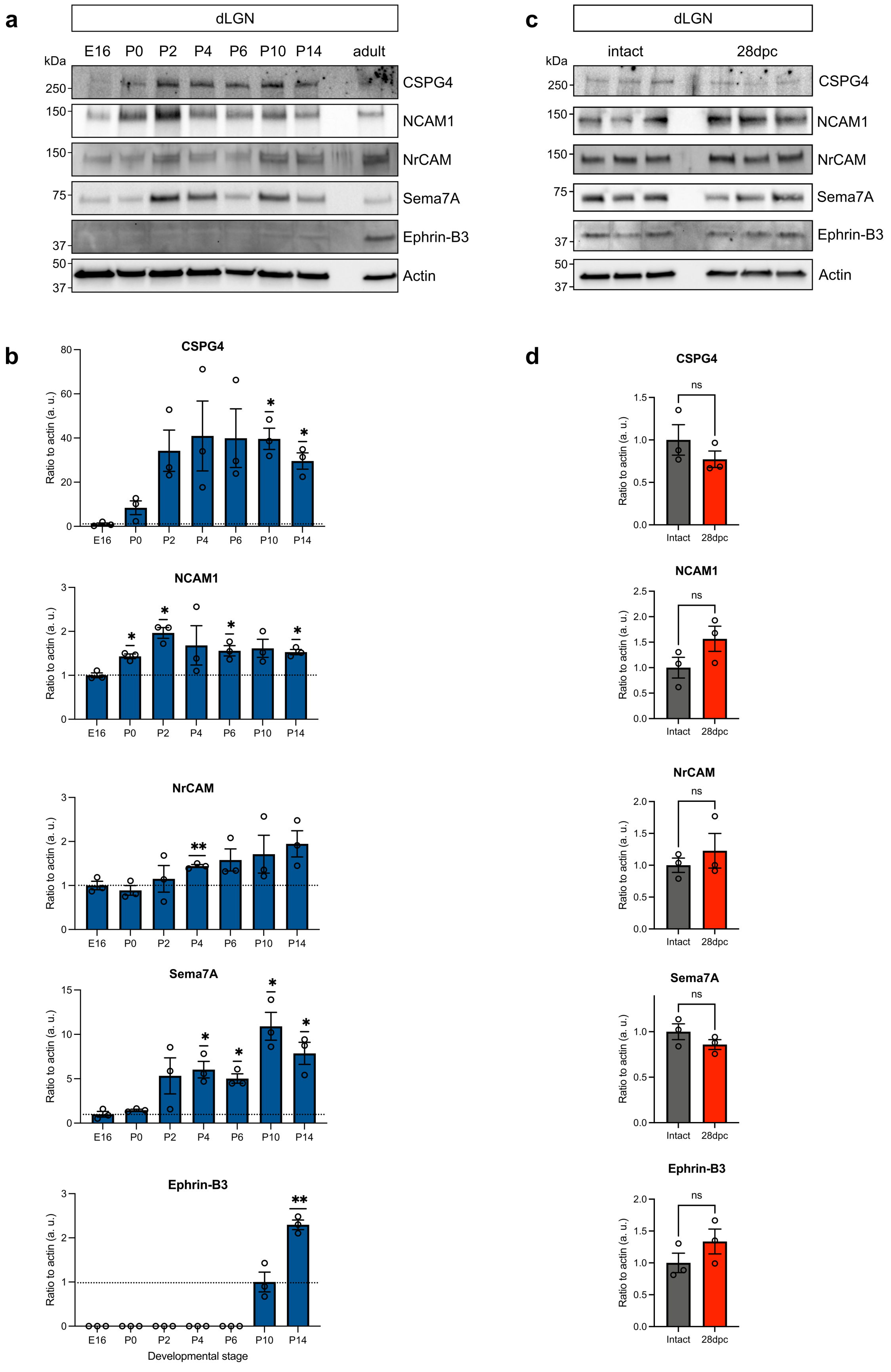

Supplementary Fig.5 Dynamic of guidance molecules expression during the dLGN development. **a** Western blot analysis of CSPG4, NCAM1, NrCAM, Sema7A and Ephrin-B3 in the dLGN during development. **b** Quantification of Western blot in **a**. N=3 biologically independent animals. Data are presented as mean values +/- SEM. Two-tailed one-sample t-tests to theoretical value = 1, * p-value < 0.05, ** p-value < 0.01. Exact p-values are provided in Source Data file. **c** Western blot analysis of CSPG4, NCAM1, NrCAM, Sema7A and Ephrin-B3 in 3 independent replicates of intact and injured (28dpc) adult dLGN. **d** Quantification of Western blot in **c**. N=3 biologically independent animals. Data are presented as mean values +/- SEM. Two-tailed unpaired Student's t-tests, ns: not significant. Source data are provided as a Source Data file.

Supplementary Figure 6

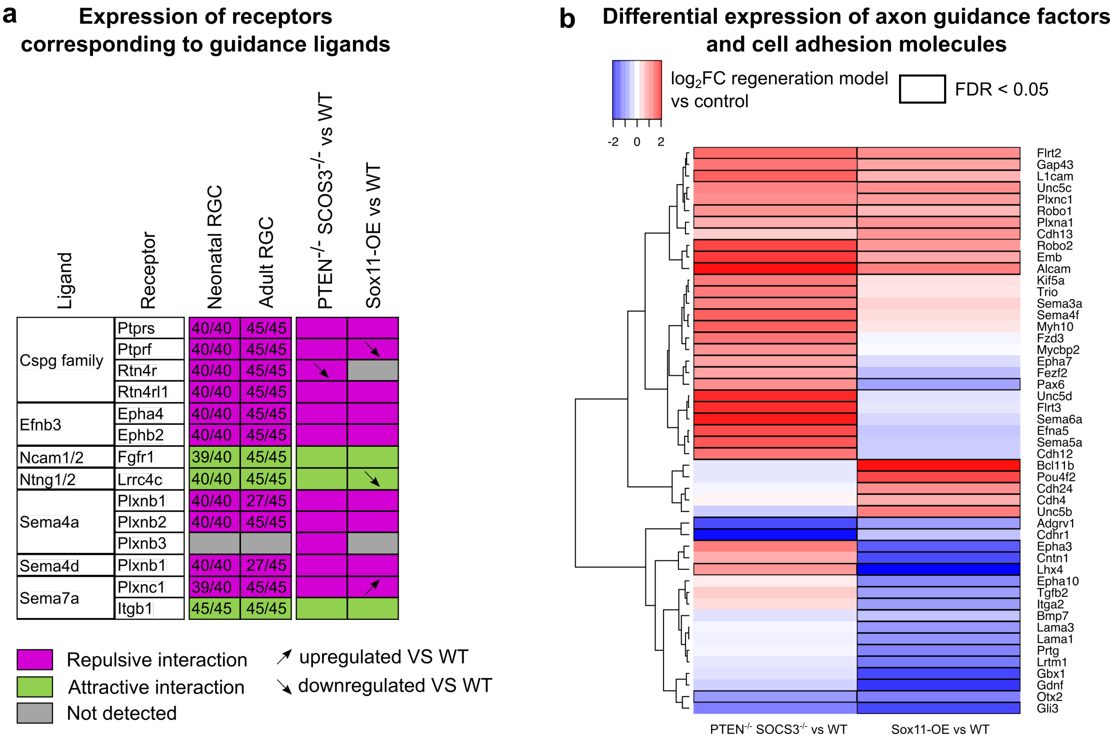

Supplementary Fig. 6 Screen of guidance factors expressed in RGC in intact and injured conditions. a Table representing the expression of guidance receptors corresponding to guidance ligands detected in adult visual targets. In red: repulsive interaction, in green: attractive interaction, in grey: not detected. For intact atlases (Neonatal RGC[1] and Adult RGC[2]), the number of RGC clusters represented is indicated in the boxes. For regenerative datasets (PTEN$^{-/-}$ SOCS3$^{-/-}$[3] and Sox11-OE[4]), the variation of the receptor compared to WT (non-regenerative) injured condition is indicated in the boxes if it is upregulated or downregulated (else steady). **b** Heatmap showing the variation of expression of guidance or cell adhesion molecules between regenerative models and WT (injured) condition (PTEN$^{-/-}$ SOCS3$^{-/-}$ and Sox11-OE). The values are $\log_2$ fold-change (FC). The genes with significant FC difference (FDR-adjusted p-value < 0.05) are boxed. Sox11-OE: Sox11-overexpressing.

Supplementary Figure 7

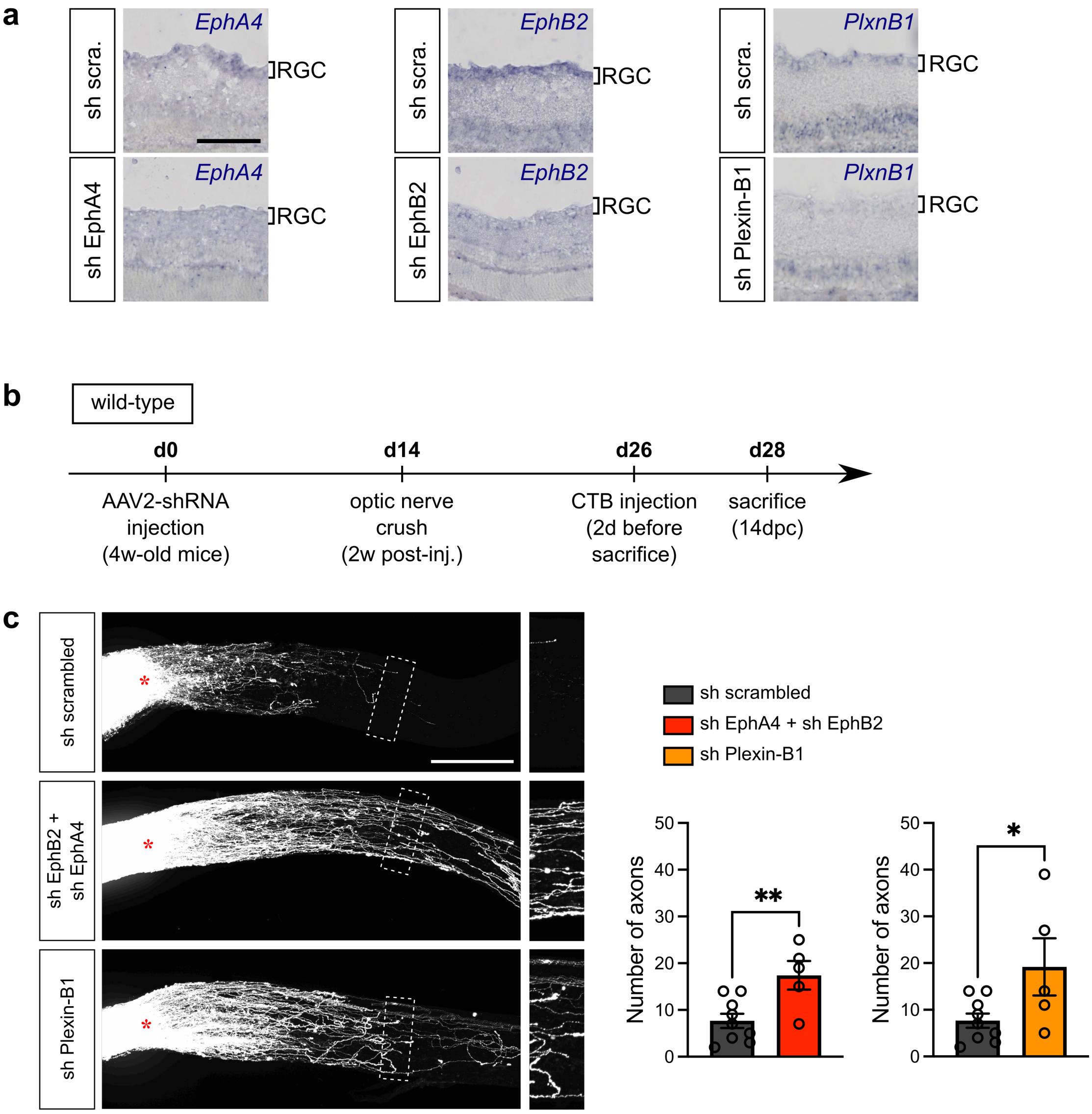

Supplementary Fig. 7 Axon regeneration in WT mice upon guidance receptors silencing. a In situ hybridization showing EphA4, EphB2, and Plexin-B1 expression in RGC of WT intact retina infected with shRNA scrambled, shRNA EphA4, shRNA EphB2 or shRNA Plexin-B1. Scale bar: 100µm. Images are representative of N=4 eyes. **b** Timeline of the experiment for WT mice. 14 days after shRNA injection, optic nerve crush was performed. 14 days post crush (14dpc), phenotypes were analysed. **c** Whole optic nerve confocal images showing axons labelled with CTB in WT mice injected with AAV2-shRNA at 14dpc, with corresponding quantification of axon number at 750µm from the injury site, indicated by a red star. Sh scrambled: N=9 biologically independent animals. Sh EphA4 + sh EphB2: N=5 biologically independent animals. Sh Plexin-B1: N=5 biologically independent animals. Data are presented as mean values +/- SEM. Two-tailed unpaired Student's t-

tests, * p-value = 0.0357, ** p-value = 0.0075. Scale bar: 250μm. Source data are provided as a Source Data file.

Supplementary References

1. Rheaume, B. A. *et al.* Single cell transcriptome profiling of retinal ganglion cells identifies cellular subtypes. *Nat Commun* **9**, (2018).
2. Tran, N. M. *et al.* Single-Cell Profiles of Retinal Ganglion Cells Differing in Resilience to Injury Reveal Neuroprotective Genes. *Neuron* **104**, 1039-1055.e12 (2019).
3. Sun, F. *et al.* Sustained axon regeneration induced by co-deletion of PTEN and SOCS3. *Nature* **480**, 372–375 (2011).
4. Norsworthy, M. W. *et al.* Sox11 Expression Promotes Regeneration of Some Retinal Ganglion Cell Types but Kills Others. *Neuron* **94**, 1112-1120.e4 (2017).
