# Supplementary material for: Guidance landscapes unveiled by quantitative proteomics to control reinnervation in adult visual system
Source: Nat Commun. 2022 Oct 13;13:6040. doi: 10.1038/s41467-022-33799-4 (PMC9561644; doi:10.1038/s41467-022-33799-4)
Supplement: Supplementary file 3 — Description of Supplementary Data [file 41467_2022_33799_MOESM3_ESM.docx]

**Description of Supplementary Files**

**File Name: Supplementary Data 1**

**Description: MS-based proteomic characterization of intact brain targets.** The analysis was based on four independent biological replicates of each RGC target. In total, this strategy allowed the identification of 6,241 proteins, with 4,186 proteins identified in the chiasm, 3,850 in the SCN, 4,612 in the vLGN, 4,834 in the dLGN and 5,097 in the SCol. SCN, suprachiasmatic nucleus; vLGN, ventral lateral geniculate nucleus; dLGN, dorsal lateral geniculate nucleus; SCol, superior colliculus.

**File Name: Supplementary Data 2**

**Description: List of proteins in each category of interest (ECM, adhesion, axon growth and guidance).** Focus on proteins identified in a minimum of three replicates of one brain (4,467 proteins out of the 6,241 proteins identified in total)

**File Name: Supplementary Data 3**

**Description: List of proteins related to axon guidance and guidance-associated factors in each visual target.** Each tab contains the proteins identified in one visual target and classified according to different categories manually defined (cell adhesion, extracellular matrix, axon growth and guidance, see **Supplementary Data 2**). Each entry is given with the protein accession number, gene name and description.

**File Name: Supplementary Data 4**

**Description: MS-based quantitative proteomic analyzes of intact brain targets or after optic nerve crush.** Up-regulated and down-regulated proteins are represented in individual tabs for each visual target. Statistical testing was conducted using limma test. Differentially-expressed proteins were sorted out using a log_2_(fold change) cut-off of 0.8 and a p-value cut-off allowing to reach a FDR inferior to 5% according to the Benjamini-Hochberg procedure.
