# Supplementary material for: Guidance landscapes unveiled by quantitative proteomics to control reinnervation in adult visual system
Source: Nat Commun. 2022 Oct 13;13:6040. doi: 10.1038/s41467-022-33799-4 (PMC9561644; doi:10.1038/s41467-022-33799-4)
Supplement: Supplementary file 5 — Reporting Summary [file 41467_2022_33799_MOESM5_ESM.pdf]

nature portfolio

Corresponding author(s): Homaira Nawabi

Last updated by author(s): Sep 20, 2022

Reporting Summary

Nature Portfolio wishes to improve the reproducibility of the work that we publish. This form provides structure for consistency and transparency in reporting. For further information on Nature Portfolio policies, see our Editorial Policies and the Editorial Policy Checklist.

Statistics

For all statistical analyses, confirm that the following items are present in the figure legend, table legend, main text, or Methods section.

n/a	Confirmed	
☐	☒	The exact sample size (*n*) for each experimental group/condition, given as a discrete number and unit of measurement
☐	☒	A statement on whether measurements were taken from distinct samples or whether the same sample was measured repeatedly
☐	☒	The statistical test(s) used AND whether they are one- or two-sided<br>*Only common tests should be described solely by name; describe more complex techniques in the Methods section.*
☒	☐	A description of all covariates tested
☐	☒	A description of any assumptions or corrections, such as tests of normality and adjustment for multiple comparisons
☐	☒	A full description of the statistical parameters including central tendency (e.g. means) or other basic estimates (e.g. regression coefficient) AND variation (e.g. standard deviation) or associated estimates of uncertainty (e.g. confidence intervals)
☐	☒	For null hypothesis testing, the test statistic (e.g. *F*, *t*, *r*) with confidence intervals, effect sizes, degrees of freedom and *P* value noted<br>*Give P values as exact values whenever suitable.*
☒	☐	For Bayesian analysis, information on the choice of priors and Markov chain Monte Carlo settings
☒	☐	For hierarchical and complex designs, identification of the appropriate level for tests and full reporting of outcomes
☒	☐	Estimates of effect sizes (e.g. Cohen's *d*, Pearson's *r*), indicating how they were calculated

Our web collection on statistics for biologists contains articles on many of the points above.

Software and code

Policy information about availability of computer code

Data collection

Proteomic data were collected as described in the Material and Methods section.
The following softwares were used:
- acquisition of MS and MS/MS data: Xcalibur 4.0 with configured instrument Q Exactive HF - Orbitrap MS 2.9 software (Thermo Scientific)
- MS-based proteomic data processing: Mascot Distiller software (version 2.7.1.0, Matrix Science)
- peptide and protein identification: Mascot (version 2.6)

Immunofluorescence data were imaged as described in the Material and Methods section.
The following softwares were used:
- lightsheet microscopy: LaVision Biotec, Imaris software (x64 version 9.9.1)
- epifluorescence microscopy on Nikon Ti2 Eclipse: NIS-Elements (version 5.11.02)
- confocal imaging on spinning disk Dragonfly (Andor): Metamorph (version 7.10.2.240)
- confocal imaging on confocal microscope LSM710 (Zeiss) and Airyscan processing: Zen (version 2.1 SP3)

Data analysis

Proteomic data were analyzed as described in the Material and Methods section.
The following softwares were used:
- proteomic data quantification: Proline software (version 2.0)
- gene ontology analysis: DAVID (Database for Annotation, Visualization and Integrated Discovery) Bioinformatics Resources (version 6.8)
- interactome analysis: STRING (version 11.0)
- Venn diagrams: Bioinfomatics and Evolutionary Genomics webtool Venn
- data analysis and representation: R software for statistical computing (version 4.0.2)
- image analysis: Fiji (version 2.0.0)

Statistical analysis was performed as described in the Material and Methods section.
GraphPad Prism (version 9.1.2) was used for statistical analysis.

For manuscripts utilizing custom algorithms or software that are central to the research but not yet described in published literature, software must be made available to editors and reviewers. We strongly encourage code deposition in a community repository (e.g. GitHub). See the Nature Portfolio guidelines for submitting code & software for further information.

Data

Policy information about availability of data

All manuscripts must include a data availability statement. This statement should provide the following information, where applicable:

- Accession codes, unique identifiers, or web links for publicly available datasets
- A description of any restrictions on data availability
- For clinical datasets or third party data, please ensure that the statement adheres to our policy

The LC-MS/MS data have been submitted to the ProteomeXchange Consortium via the PRIDE partner repository under dataset identifier PXD029325 [http://proteomecentral.proteomexchange.org/cgi/GetDataset?ID=PXD029325]. For peptide and protein identification with Mascot, concomitant searches were done against Uniprot database [https://www.uniprot.org/] (Mus Musculus taxonomy, for Optic chiasm and SCN samples, downloaded in June 2019 with 87573 entries, and for dlGN, vLGN and SCol samples, downloaded in August 2020 with 87975 entries). The protein expression data generated in this study are available in Supplementary Data 1-4. The Western blot data generated in this study are provided in the Source Data file. Quantification and statistics of source data are provided in the Source Data file. Source data are provided with this paper. RGC gene expression data used in this study are available on NCBI's GEO database under the accession codes: GSE115404 [https://www.ncbi.nlm.nih.gov/geo/query/acc.cgi?acc=GSE115404] (atlas of neonatal (P5) RGC from single-cell transcriptomics analysis, user-friendly version at https://health.uconn.edu/neuroregeneration-lab/rgc-subtypes-gene-browser/); GSE137400 [https://www.ncbi.nlm.nih.gov/geo/query/acc.cgi?acc=GSE137400] (atlas of adult RGC from single-cell transcriptomics analysis, user-friendly version at https://singlecell.broadinstitute.org/single_cell/study/SCP509/mouse-retinal-ganglion-cell-adult-atlas-and-optic-nerve-crush-time-series/); GSE32309 [https://www.ncbi.nlm.nih.gov/geo/query/acc.cgi?acc=GSE32309] (microarray dataset comparing PTEN-/- SOCS3-/- RGC to WT RGC after optic nerve crush); GSE87046 [https://www.ncbi.nlm.nih.gov/geo/query/acc.cgi?acc=GSE87046] (RNA-sequencing dataset comparing Sox11-overexpressing RGC to control RGC after optic nerve crush).

Human research participants

Policy information about studies involving human research participants and Sex and Gender in Research.

Reporting on sex and gender	Not applicable
Population characteristics	Not applicable
Recruitment	Not applicable
Ethics oversight	Not applicable

Note that full information on the approval of the study protocol must also be provided in the manuscript.

Field-specific reporting

Please select the one below that is the best fit for your research. If you are not sure, read the appropriate sections before making your selection.

☒ Life sciences ☐ Behavioural & social sciences ☐ Ecological, evolutionary & environmental sciences

For a reference copy of the document with all sections, see nature.com/documents/nr-reporting-summary-flat.pdf

Life sciences study design

All studies must disclose on these points even when the disclosure is negative.

Sample size

For proteomic experiments, sample size (n=4 independent biological replicates per brain region and per condition) was chosen in agreement with common practices in the field (eg Fusco et al., Nature Communications, 2021, https://doi.org/10.1038/s41467-021-26365-x).
For biochemical validation (Western blot experiments), sample size (n=3 independent biological replicates) was chosen in agreement with common practices in the field (eg Fusco et al., Nature Communications, 2021, https://doi.org/10.1038/s41467-021-26365-x).
For ex vivo experiments (stripe assays), sample size (n=7-10 individual explants) was chosen based on preliminary data and on common practices in the field (eg Catlett et al., Nature Communications, 2021, https://doi.org/10.1038/s41467-021-22770-4).
For phenotypic experiments, sample size (n=3-9 mice per group) was chosen in agreement with common practices in the field and with respect to the 3R rule (replace, reduce, refine) (eg Lu et al., Nature, 2020, https://doi.org/10.1038/s41586-020-2975-4).
All attempts of replication were successful.

Data exclusions

For ex vivo experiments, explant cultures that did not grow were excluded from the analysis.
For phenotypic experiments in wild-type mice, optic nerves with incomplete crush or poorly perfused optic nerves were excluded from the analysis.
For phenotypic experiments in the long-distance regeneration model, optic nerves were excluded if: crush was incomplete; perfusion was

	poor; regeneration was insufficient (not reaching the chiasm); or regeneration was overextended, ie not matching the initial publication (Sun et al., Nature, 2011).
Replication	For proteomic analysis and biochemical experiments, samples were collected from biologically independent animals and performed once.<br>For ex vivo experiments, individual explants were treated as biologically independent replicates. Two independent experiments (days of culture) were performed.<br>For phenotypic experiments, biologically independent animals over one or two experiments (days of surgery) were considered.
Randomization	For ex vivo experiments, retina explants were obtained from one mouse on each day of culture and were randomly assigned to a patterned stripe.<br>For phenotypic experiments, mice were randomly assigned to each experimental group.
Blinding	For ex vivo and in vivo experiments, researchers were not blinded to avoid mistakes when performing the experiment.<br>For data collection and analysis, researchers were blinded by allocating random numbers to individual samples.

Reporting for specific materials, systems and methods

We require information from authors about some types of materials, experimental systems and methods used in many studies. Here, indicate whether each material, system or method listed is relevant to your study. If you are not sure if a list item applies to your research, read the appropriate section before selecting a response.

Materials & experimental systems

n/a	Involved in the study
☐	☒ Antibodies
☒	☐ Eukaryotic cell lines
☒	☐ Palaeontology and archaeology
☐	☒ Animals and other organisms
☒	☐ Clinical data
☒	☐ Dual use research of concern

Methods

n/a	Involved in the study
☒	☐ ChIP-seq
☒	☐ Flow cytometry
☒	☐ MRI-based neuroimaging

Antibodies

Antibodies used	For biochemical experiments (Western blot analysis), the following primary antibodies were used:<br>anti-NCAM1 (1:1000, Rabbit, Cell signalling Technology, #99746), anti-Tenascin-C (1:1000, Rabbit, Abcam, ab108930), anti-Sema4D (1:1000, Rabbit, Abcam, ab134128), anti-Sema7A (1:1000, Rabbit, Abcam, ab23578), anti-Neuropilin-2 (1:1000, Rabbit, Cell signalling Technology, #3366), anti-Plexin-A4 (1:1000, Rabbit, Cell signalling Technology, #3816), anti-Ephrin-B3 (1:500, Rabbit, Invitrogen, 34-3600), anti-CSPG4 (1:1000, Rabbit, Proteintech, 55027-1-AP), anti-NrCAM (1:1000, Rabbit, Abcam, ab24344), anti-actin (1:5000, Mouse, Sigma-Aldrich, a1978).<br><br>For biochemical experiments (Western blot analysis), the following secondary antibodies were used:<br>horseradish peroxidase-conjugated secondary antibody: anti-rabbit (1:5000, Proteintech, SA00001-2) or anti-mouse (1:10000, ThermoFisher Scientific, A16011).<br><br>For immunostaining experiments, the following primary antibodies were used:<br>anti-NCAM1 (1:100, Rabbit, Cell signalling Technology, #99746), anti-DCLK2 (1:100, Rabbit, Abcam, ab106639), anti-Sema4D (1:100, Rabbit, Abcam, ab134128), anti-CSPG4 (1:100, Rabbit, Proteintech, 55027-1-AP), anti-Ephrin-B3 (1:100, Mouse, R&D Systems, MAB395), anti-GFAP (1:200, Rat, Thermo Fisher Scientific, 13-0300), anti-Sema7A (1:100, Rabbit, Abcam, ab23578), anti-NrCAM (1:100, Rabbit, Abcam, ab24344), anti-Olig2 (1:100, Goat, R&D Systems, AF2418), anti-Iba1 (1:100, Goat, Novus Biologicals, NB100-1028), anti-NeuN (1:100, Mouse, Sigma-Aldrich, MAB377), anti-NeuN (1:100, Rabbit, Abcam, ab177487), anti-EphB2 (1:200, Rabbit, Abcam, ab216629), anti-EphA4 (1:200, Rabbit, Thermo Fisher Scientific, 21875-1-AP), anti-Plexin-B1 (1:200, Mouse, R&D Systems, MAB3749), anti-Plexin-C1 (1 :200, Mouse, R&D Systems, AF5375) and anti-β-tubulin III (TUJ1, 1:500, Mouse, Biolegend, 801202 or Rabbit, Abcam, ab18207).<br><br>For immunostaining experiments, the following secondary antibodies were used:<br>Alexa-fluor 488 conjugated anti-rabbit (1:200, ThermoFisher Scientific, A21206), Alexa-fluor 568 conjugated anti-rabbit (1:200, ThermoFisher Scientific, A10042), Alexa-fluor 647 conjugated anti-rabbit (1:200, ThermoFisher Scientific, A31573), Alexa-fluor 488 conjugated anti-mouse (1:200, ThermoFisher Scientific, A21202), Alexa-fluor 568 conjugated anti-mouse (1:200, ThermoFisher Scientific, A11004), Alexa-fluor 647 conjugated anti-mouse (1:200, ThermoFisher Scientific, A31571), Alexa-fluor 488 conjugated anti-rat (1:200, Jackson Laboratory, 712-545-150), Alexa-fluor 647 conjugated anti-rat (1:200, Jackson Laboratory, 712-605-150), Alexa-fluor 488 conjugated anti-goat (1:200, ThermoFisher Scientific, A11055), Alexa-fluor 568 conjugated anti-goat (1:200, ThermoFisher Scientific, A11057).
Validation	- anti-NCAM1 (Rabbit, Cell signalling Technology, #99746), immunohistochemistry and Western blot, validated by the manufacturer and in peer-reviewed literature (https://www.cellsignal.com/products/primary-antibodies/ncam1-cd56-e7x9m-xp-rabbit-mab/99746)<br>- anti-Tenascin-C (Rabbit, Abcam, ab108930), Western blot, validated by the manufacturer and in peer-reviewed literature (https://www.abcam.com/tenascin-c-antibody-epr4219-ab108930.html)<br>- anti-Sema7A (Rabbit, Abcam, ab23578), immunohistochemistry and Western blot, validated by the manufacturer and in peer-reviewed literature (https://www.abcam.com/semaphorin-7a-antibody-ab23578.html)

-anti-Neuropilin-1 (Rabbit, Cell signalling Technology, #3725), Western blot, validated by the manufacturer and in peer-reviewed literature (https://www.cellsignal.com/products/primary-antibodies/neuropilin-1-d62c6-rabbit-mab/3725)
- anti-Neuropilin-2 (Rabbit, Cell signalling Technology, #3366), Western blot, validated by the manufacturer and in peer-reviewed literature (https://www.cellsignal.com/products/primary-antibodies/neuropilin-2-d39a5-xp-rabbit-mab/3366)
- anti-Plexin-A4 (Rabbit, Cell signalling Technology, #3816), Western blot, validated by the manufacturer and in peer-reviewed literature (https://www.cellsignal.com/products/primary-antibodies/plexin-a4-c5d1-rabbit-mab/3816)
- anti-Ephrin-B3 (Rabbit, Invitrogen, 34-3600), immunofluorescence and Western blot, validated by the manufacturer and in peer-reviewed literature (https://www.thermofisher.com/antibody/product/Ephrin-B3-Antibody-Polyclonal/34-3600)
- anti-CSPG4 (Rabbit, Proteintech, 55027-1-AP), immunohistochemistry and Western blot, validated by the manufacturer and in peer-reviewed literature (https://www.ptglab.com/products/CSPG4,NG2-Antibody-55027-1-AP.htm)
- anti-NrCAM (Rabbit, Abcam, ab24344), immunohistochemistry and Western blot, validated by the manufacturer and in peer-reviewed literature (https://www.abcam.com/nrcam-antibody-neuronal-marker-ab24344.html)
- anti-actin (Mouse, Sigma-Aldrich, a1978), Western blot, validated by the manufacturer and in peer-reviewed literature (https://www.sigmaaldrich.com/FR/fr/product/sigma/a1978)
- anti-DCLK2 (Rabbit, Abcam, ab106639), immunohistochemistry, validated by the manufacturer and in peer-reviewed literature (https://www.abcam.com/dclk2-antibody-ab106639.html)
- anti-Sema4D (Rabbit, Abcam, ab134128), Western blot and immunohistochemistry, validated by the manufacturer and in peer-reviewed literature (https://www.abcam.com/semaphorin-4dcd100-antibody-ep3569-ab134128.html)
- anti-GFAP (Rat, Thermo Fisher Scientific, 13-0300), immunofluorescence, validated by the manufacturer and in peer-reviewed literature (https://www.thermofisher.com/antibody/product/GFAP-Antibody-clone-2-2B10-Monoclonal/13-0300)
- anti-Olig2 (Goat, R&D Systems, AF2418), immunofluorescence, validated by the manufacturer and in peer-reviewed literature (https://www.rndsystems.com/products/human-mouse-rat-olig2-antibody_af2418)
- anti-Iba1 (Goat, Novus Biologicals, NB100-1028), immunofluorescence, validated by the manufacturer and in peer-reviewed literature (https://www.novusbio.com/products/aif-1-iba1-antibody_nb100-1028)
- anti-NeuN (Mouse, Sigma-Aldrich, MAB377), immunofluorescence, validated by the manufacturer and in peer-reviewed literature (https://www.sigmaaldrich.com/FR/fr/product/mm/mab377)
- anti-NeuN (Rabbit, Abcam, ab177487), immunofluorescence, validated by the manufacturer and in peer-reviewed literature (https://www.abcam.com/neun-antibody-epr12763-neuronal-marker-ab177487.html)
- anti-EphB2 (Rabbit, Abcam, ab216629), immunohistochemistry, validated by the manufacturer (https://www.abcam.com/eph-receptor-b2-antibody-ab216629.html)
- anti-EphA4 (Rabbit, Thermo Fisher Scientific, 21875-1-AP), immunofluorescence, validated by the manufacturer and in peer-reviewed literature (https://www.thermofisher.com/antibody/product/EPHA4-Antibody-Polyclonal/21875-1-AP)
- anti-Plexin-B1 (Mouse, R&D Systems, MAB3749), immunohistochemistry, validated by the manufacturer and in peer-reviewed literature (https://www.rndsystems.com/products/human-plexin-b1-antibody-439512_mab3749)
- anti-Plexin-C1 (Mouse, R&D Systems, AF5375), immunohistochemistry, validated by the manufacturer and in peer-reviewed literature (https://www.rndsystems.com/products/mouse-plexin-c1-antibody_af5375)
- anti-β-tubulin III (TUJ1, Mouse, Biolegend, 801202), immunofluorescence, validated by the manufacturer and in peer-reviewed literature (https://www.biolegend.com/ja-jp/products/purified-anti-tubulin-beta-3-tubb3-antibody-11580)
- anti-β-tubulin III (Rabbit, Abcam, ab18207), immunofluorescence, validated by the manufacturer and in peer-reviewed literature (https://www.abcam.com/beta-iii-tubulin-antibody-neuronal-marker-ab18207.html)

Animals and other research organisms

Policy information about studies involving animals; ARRIVE guidelines recommended for reporting animal research, and Sex and Gender in Research

Laboratory animals	Wild-type (WT) embryos (E16.5), pups (P0, P2, P4, P6, P10, P14) and adult (6 to 10 week-old) mice were used in this study, regardless of their sex.<br>In all experiments, Ptenfl/fl and Pten fl/fl SOCS3fl/fl mouse lines were used as wild-type if mice did not receive AAV2-Cre injection. PTEN fl/fl SOCS3 fl/fl mice were used for in vivo and co-culture experiments.<br>All mice were housed in standard housing conditions with a 12h light/dark cycle. Where possible, mice were housed in groups of 2-5 per cage. Mice were fed and watered ad libitum.
Wild animals	No wild animals were used in this study.
Reporting on sex	Mice were allocated to experimental groups regardless of their sex.
Field-collected samples	No field collected samples for this study.
Ethics oversight	All the in vivo experiments were performed in accordance with our ethics protocol approved by the institution, local ethics committee and the French and European guidelines (Ministère de l'Enseignement Supérieur, de la Recherche et de l'Innovation, APAFIS#9145-201612161701775v3 and APAFIS#26565-2020061613307385v3).

Note that full information on the approval of the study protocol must also be provided in the manuscript.
